# Chemical Evaluation of Eumelanin Maturation by ToF-SIMS and Alkaline Peroxide Oxidation HPLC Analysis

**DOI:** 10.3390/ijms22010161

**Published:** 2020-12-26

**Authors:** Martin Jarenmark, Peter Sjövall, Shosuke Ito, Kazumasa Wakamatsu, Johan Lindgren

**Affiliations:** 1 Department of Geology, Lund University, 223 62 Lund, Sweden; johan.lindgren@geol.lu.se; 2 The Materials and Production Division, RISE Research Institutes of Sweden, 501 15 Borås, Sweden; 3 Institute for Melanin Chemistry, Fujita Health University, Toyoake, Aichi 470-1192, Japan; sito@fujita-hu.ac.jp (S.I.); kwaka@fujita-hu.ac.jp (K.W.)

**Keywords:** alkaline hydrogen peroxide oxidation, eumelanin, maturation, principal component analysis, time-of-flight secondary ion mass spectrometry

## Abstract

Residual melanins have been detected in multimillion-year-old animal body fossils; however, confident identification and characterization of these natural pigments remain challenging due to loss of chemical signatures during diagenesis. Here, we simulate this post-burial process through artificial maturation experiments using three synthetic and one natural eumelanin exposed to mild (100 °C/100 bar) and harsh (250 °C/200 bar) environmental conditions, followed by chemical analysis employing alkaline hydrogen peroxide oxidation (AHPO) and time-of-flight secondary ion mass spectrometry (ToF-SIMS). Our results show that AHPO is sensitive to changes in the melanin molecular structure already during mild heat and pressure treatment (resulting, e.g., in increased C-C cross-linking), whereas harsh maturation leads to extensive loss of eumelanin-specific chemical markers. In contrast, negative-ion ToF-SIMS spectra are considerably less affected by mild maturation conditions, and eumelanin-specific features remain even after harsh treatment. Detailed analysis of ToF-SIMS spectra acquired prior to experimental treatment revealed significant differences between the investigated eumelanins. However, systematic spectral changes upon maturation reduced these dissimilarities, indicating that intense heat and pressure treatment leads to the formation of a common, partially degraded, eumelanin molecular structure. Our findings elucidate the complementary nature of AHPO and ToF-SIMS during chemical characterization of eumelanin traces in fossilized organismal remains.

## 1. Introduction

Melanins are a group of natural pigments (biochromes) found in a wide variety of organisms. They are among the most abundant biochromes in the animal kingdom (including us humans), where they serve a multitude of functions in different anatomies and organ systems. These include UV protection in skin, optical screening in eyes, and coloration of hairs and feathers [1,2,3]. In vertebrates, the most common types of melanins are eumelanin and pheomelanin, which are associated with black-brown and red-yellow colors, respectively. Interestingly, eumelanin (and possibly also pheomelanin) has been found to persist across geological time, providing biomolecular data from multimillion-year-old fossils [4,5,6,7,8,9,10]. This information can be used to infer novel biological, ecological, and evolutionary aspects of now long-extinct animals, including traits associated with crypsis, thermoregulation, and social interactions [4,5,6,7,8].

**Figure 1:**
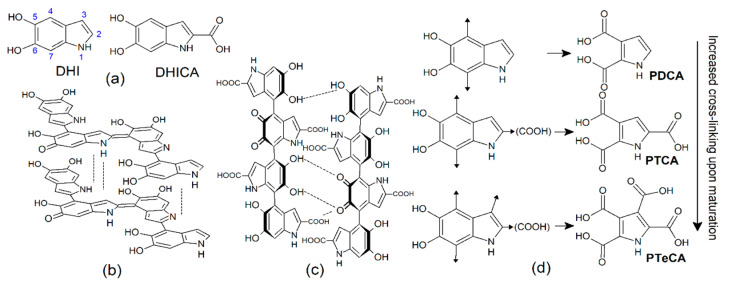
(**a**) Structures of DHI and DHICA, the two monomer units used to produce eumelanin, with IUPAC numbering scheme for indole units in blue. (**b**) Example of a eumelanin oligomer structure made solely from DHI, as proposed by d’Ischia et al., 2015, and Micillo et al., 2017, [3,16]. (**c**) Proposed structure [17] of a eumelanin polymer backbone made entirely from DHICA. Note that the ring system of each monomer unit is out of plane relative to the adjacent ones. (**d**) Various units in the proposed structure of DHI and DHICA eumelanins, together with the corresponding degradation products generated when these pigments are subjected to alkaline hydrogen peroxide oxidation (AHPO) (adapted from Ito et al., 2013, [18]).

In vivo, melanins are produced in a multistep process, which initially comprises oxidation of the amino acid tyrosine followed by comprehensive polymerization [3]. Due to their refractory and non-soluble properties, melanins are difficult to examine with most standard analytical techniques. As a consequence, the detailed molecular structure of, e.g., eumelanin, remains incompletely known [11]. It has been established though that the polymeric structure of natural eumelanin is composed of two monomer units, 5,6-dihyroxyindole (DHI) and 5,6-dihydroxyindole-2-carboxylic acid (DHICA) (Figure 1a), occurring in sub-equal proportions [12]. However, the precise configuration in which they are bound to each other is not fully understood, and may deviate in different species, organs and/or tissues [3]. Model studies based on synthetic eumelanins and various oligomers of DHI and DHICA have indicated that these two monomers may be coupled to one another as indicated in Figure 1b,c. Due to electronic and steric factors, the DHI units typically form roughly planar oligomers connected primarily at the 2,2′-, 2,4′-, and 2,7′-position of the indole moiety (Figure 1a). DHICA polymerization is affected by the presence of a carboxylic acid group at the 2-position, which decreases nucleophilicity of the pyrrole moiety through its electron-withdrawing nature, thereby directing reactivity mainly towards the 4,4′-, 4,7′-, and 7,7′-bonding formation, with lower involvement of the 3-position [13,14,15,16,17].

The aggregation of those oligomers formed during the initial stages of the oxidative polymerization of DHI and DHICA has been examined under scanning (SEM) and transmission electron microscopy (TEM) [19]. DHI melanin forms globular, almost onion-like aggregates with a diameter of about 50 nm via π-stacking with high intramolecular π-electron delocalization after polymerization of the DHI unit (Figure 1b). On the other hand, SEM and TEM images of DHICA polymers revealed relatively large and elongate structures more than 100 nm long [19]. The peculiar properties of DHICA melanin are controlled by a carboxylate group, which forces the inter-ring dihedral angles to twist, thereby minimizing electrostatic interactions. The resulting oligoindole chains are not amenable to π-stacking, but instead form the rod-like shape (>100 nm) after bundling by conformationally hindered and interrupted π-conjugation (Figure 1c) [3,16].

For mixed melanins, DHI and DHICA likely are present at different proportions depending on reaction conditions [20]. While this chemically disordered model is thought to account for much of the unique properties of eumelanin, geometric constraints and inter-oligomer bonding configurations likely also play important roles [21]. It is noteworthy that these monomer units also can connect at the 3-position of the indole ring [22], although at a lower degree than during the early stages of melanogenesis [1]. However, it has been shown that this type of cross-linking increases upon aging and maturation of eumelanin [18,23].

One of the most powerful and well-established methods to identify, quantify, and characterize melanins in biological samples is based on treatment with alkaline hydrogen peroxide, followed by the detection of a set of unique degradation products by HPLC [20,24] (Figure 1d). For eumelanins, two of the most important degradation products are PDCA (pyrrole-2,3-dicarboxylic acid) and PTCA (pyrrole-2,3,5-tricarboxylic acid). These two compounds largely represent DHI and DHICA, respectively, in the eumelanin molecular structure, although PTCA also stems from DHI cross-linked at the 2-position. More recently, it has further been discovered that cross-linking at the 3-position of the indole structure can be quantified by detection of two additional degradation products: PTeCA (pyrrole-2,3,4,5-tetracarboxylic acid; Figure 1d) and iso-PTCA (pyrrole-2,3,4-tricarboxylic acid) [18]. PTeCA is thought to reflect the concentration of DHI units cross-linked in positions 2 and 3 or DHICA cross-linked in position 3.

During the last decade, alkaline hydrogen peroxide oxidation (AHPO) has increasingly been used to detect and quantify eumelanin traces in fossil samples [4,6,23,25,26], including squid ink sacs more than 180 million years old [6,23]. There are, however, substantial challenges related to the analysis of this pigment in ancient samples using chromatography-based techniques. These include, e.g., difficulties in extracting the molecule intact enough for chemical analysis and, perhaps more importantly, the relatively large sample quantities required for this type of analysis (especially when considering that well-preserved fossils typically are rare, and the amount of material available for destructive analysis is exceedingly small).

Time-of-flight secondary ion mass spectrometry (ToF-SIMS) is a surface analysis technique that has been extensively employed to investigate the organic content of diverse samples, including pigments in historic paintings [27] and carbonaceous traces in meteorites [28], as well as eumelanins preserved in geologic samples [4,5,6,7,10,25,29,30]. A major advantage of ToF-SIMS when analyzing fossils is that specific structures can be molecularly characterized in situ with a spatial resolution in the micrometer range. Furthermore, the technique is virtually non-destructive and minimally intrusive (at least for samples that can be fitted in the vacuum chamber of the instrument). The analysis principle includes bombardment of the sample surface with high-energy ions in a focused beam, and collection of mass spectra of those (secondary) ions that are emitted in the collision process. Scanning of the (primary) ion beam over a selected analysis region allows imaging of specific ions, or the generation of mass spectra from selected structures within the investigated area. Whereas molecular species weighing up to about 2000 Da normally can be detected as intact molecular ions, macromolecular compounds, such as melanins, instead are identified by a distinct set of fragment ions. For eumelanins, identification is based on a characteristic peak pattern in the mass range *m/z* 45–170 in negative ion spectra, corresponding to various C-, H-, N-, and O-containing fragment ions [7]. However, it is unknown how and to what degree the yields of different ions are influenced by changes in the eumelanin molecular structure that may occur during diagenetic alterations (but see also Colleary et al., 2015 [10]).

The aim of this work is to compare the AHPO and ToF-SIMS methods with regards to capabilities in detecting and characterizing eumelanins. Moreover, it will be deduced how and in what way the results from these techniques are affected by experimental maturation at both high temperatures and pressures (used as proxies for late diagenetic influence [31,32]). We used synthetic eumelanins made from DHI, DHICA, and a DHI + DHICA (1:1) mixture, as well as natural eumelanin obtained from the cephalopod *Sepia officinalis*, since these all are well-characterized model compounds [19]. We exposed all pigments to “mild” and “harsh” maturation conditions, and the results of our efforts indicate clear differences between the two methods, with AHPO demonstrating higher sensitivity to minor changes in the eumelanin structure, and ToF-SIMS being able to identify eumelanin even after extensive degradation.

## 2. Results

### 2.1. Maturation

Three synthetic eumelanins (made from (i) pure DHI; (ii) a DHI + DHICA (1:1) mixture; and (iii) pure DHICA) and one natural eumelanin (obtained from *Sepia officinalis*) were analyzed in their untreated state and following artificial maturation according to one of two protocols: (i) 100 °C/100 bar for 24 h (“mild” conditions, M) and (ii) 250 °C/250 bar for 72 h (“harsh” conditions, H). Significant mass loss was observed for all samples upon increased heat and pressure treatment (Table 1), with the highest mass losses recorded after harsh maturation. It is assumed that this loss stemmed from gaseous species, since traces of liquids or melted materials were not detected in any of the reaction vessels.

### 2.2. AHPO Analysis

**Figure 2:**
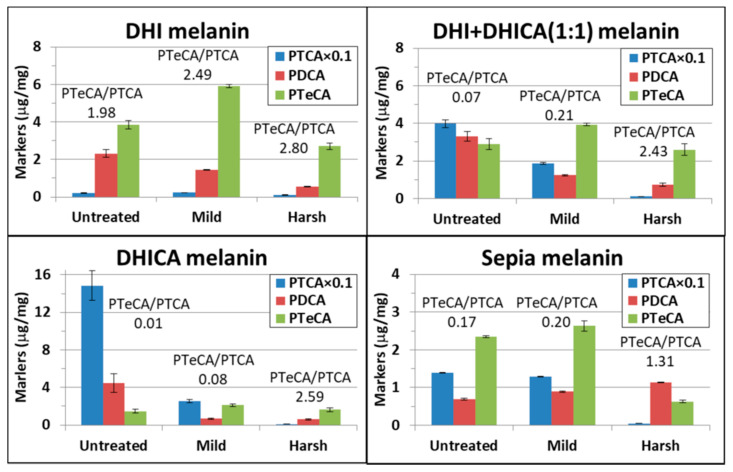
Yields of the eumelanin degradation products upon AHPO in DHI, DHI + DHICA (1:1), DHICA, and *Sepia* eumelanin after mild (100 °C/100 bar for 24 h) and harsh (250 °C/200 bar for 72 h) treatment, respectively, together with the initial amounts in the corresponding untreated samples. Presented values represent averages ± SD of two independent measurements. For visualization purposes, PTCA values have been scaled down by a factor of 10. For each maturation stage, the PTeCA/PTCA ratio is shown in the graphs.

The results from our AHPO analysis demonstrate noticeable changes in the eumelanin molecular structure upon maturation (Figure 2). For DHI melanin, mild maturation leads to an increased PTeCA concentration, suggesting amplified cross-linking of DHI at position 3, as well as a relatively small decrease in PDCA, and essentially unchanged PTCA levels. Harsh maturation results in reduced concentrations of PDCA, PTCA, and PTeCA, indicating loss of (AHPO-active) DHI structures in the pigment.

For the DHI + DHICA (1:1) melanin, the most evident effect of maturation is the reduced concentration of PTCA, from 40 µg/mg in the untreated sample to 19 µg/mg after mild maturation, and about 1 µg/mg after harsh maturation. This indicates partial and complete loss of DHICA after mild and harsh maturation, respectively. The conspicuous PTCA decrease after maturation may reflect loss of the carboxyl group in the DHICA molecular structure; however, the simplest decarboxylation mechanism, where the carboxyl group is replaced by a hydrogen atom, would transform DHICA into DHI, and thus an increased concentration of PDCA would be expected as a result of maturation. This, however, is not observed. The reduced PTCA levels may therefore indicate additional modifications of the DHICA structure in the eumelanin polymer. The low PDCA and PTCA levels observed after harsh maturation suggest nearly complete loss of both (AHPO-active) DHI and DHICA units in the eumelanin structure. Finally, the small enrichment in PTeCA concentration after mild maturation is consistent with a limited increase of cross-linking at position 3 in DHI and/or DHICA [18].

Maturation of DHICA melanin resulted in a distinct decrease of the PTCA concentration, in similarity with the condition of the DHI + DHICA (1:1) melanin but from an initially higher level (consistent with the higher relative DHICA concentration in DHICA melanin). Mild maturation of DHICA melanin is also accompanied by a clear decrease of the PDCA concentration. A small increase of the PTeCA concentration upon mild maturation may indicate limited cross-linking at position 3 of DHICA and/or the formed DHI units. As for the DHI and DHI + DHICA (1:1) melanins, harsh maturation resulted in low levels for all these three AHPO degradation products.

Upon mild maturation, the natural (*Sepia*) eumelanin displayed a relatively small decrease of PTCA, from a lower initial level relative to the DHI + DHICA (1:1) and DHICA melanins. In contrast, a significant increase in PTeCA was observed, whereas the PDCA concentration remained largely unaffected. After harsh maturation, PTCA almost completely disappeared, whereas PDCA remained seemingly unchanged and PTeCA decreased to one-fourth of that observed after mild maturation.

Comparison of the AHPO results for the different untreated eumelanin samples demonstrated that the relative concentration of DHICA is well represented by the detected concentrations of PTCA, from 150 µg/mg in the pure DHICA melanin to 40 µg/mg in the DHI + DHICA (1:1) melanin, and about 2 µg/mg in the pure DHI melanin. However, the detected PDCA concentrations do not appear to quantitatively reflect the proportion of DHI units in a comparable manner, since the measured PDCA concentrations are within the same range in the DHI, DHI + DHICA (1:1) and DHICA melanins. These results may indicate that a small amount of PDCA is produced also from DHICA in the eumelanin structure, or that a minor fraction of the DHICA units are transformed into DHI during the polymerization process. Furthermore, the detection of PTeCA in all three untreated eumelanins suggests the presence of cross-linked DHI (and DHICA) units at the 3-position in the eumelanin structures even before maturation [18].

The amounts of all AHPO markers were reduced upon harsh maturation. The most distinct decrease was observed for PTCA, whereas PDCA and PTeCA were less affected. This results in a clear increase of the PTeCA/PTCA ratio: e.g., from 0.07 in the untreated DHI + DHICA (1:1) melanin to 2.43 after harsh maturation.

### 2.3. ToF-SIMS

**Figure 3:**
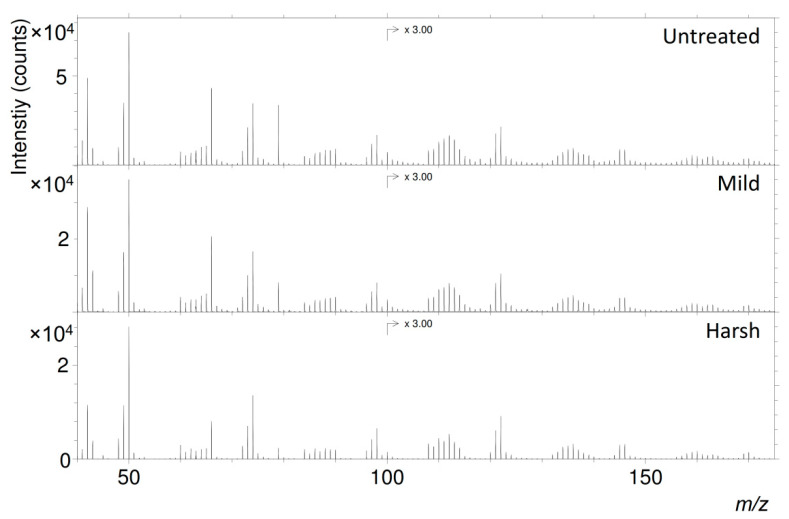
Negative-ion time-of-flight secondary ion mass spectrometry (ToF-SIMS) spectra of DHI + DHICA (1:1) melanin, untreated and after mild and harsh maturation, respectively. Specific ion assignments are listed in Table 2 and spectra for the DHI and DHICA melanins can be found in the Supplementary materials Figure S1.

Negative-ion ToF-SIMS spectra for all untreated samples showed features characteristic of eumelanins [7], exemplified by the DHI + DHICA (1:1) melanin illustrated in Figure 3 (see also Supplementary Figure S1). Following experimental maturation, all eumelanin-related peaks were retained in the spectra, and only minor changes in the relative signal intensity distribution occurred (Figure 3). Similarly, only minor differences could be observed between spectra from the various investigated eumelanins (Supplementary Figure S1). These relatively small spectral variations are in stark contrast to our AHPO results, which showed considerable differences between the various samples (Figure 2). Thus, our results indicate that AHPO is more sensitive than ToF-SIMS to molecular changes occuring upon maturation, as well as to structural differences between the various eumelanin types. However, the retained eumelanin-specific features in our ToF-SIMS spectra, even after the samples had experienced severe pressure and temperature conditions, demonstrate that the organic matter still can be recognized as eumelanin, or at least originating from this pigment, after maturation.

Despite the superficial similarity between the acquired eumelanin spectra, detailed evaluation of the signal intensities revealed systematic changes that occurred upon maturation, but also between the different pigment types. Ion assignments of the major peaks in eumelanin spectra show a repetitive pattern, where ions of different categories are repeated with intervals of *m/z* 24. This occurs throughout the entire mass range shown in Figure 3 (*m/z* 40–170); see also Table 2. Displayed categories represent different types of ions, such as C_2n_^−^, C_2n_H^−^, C_2n−1_N^−^, C_2n_HO^−^, and C_2n−1_NO^−^, and the *m/z* 24 separation corresponds to an addition of C_2_ to the previous ion. Although the mechanism by which these ions are formed during ToF-SIMS analysis is relatively complex, it can be assumed that there is close relation between the ion composition and local molecular structure from which the ion originates.

Principal component analysis (PCA) was employed to identify variations between the acquired spectra [33]. Briefly, PCA identifies peaks (i.e., original variables) that vary in signal intensity in a correlated manner between the analyzed spectra, and then combine them into a new set of variables termed principle components (PCs). The first principal component (PC1) accounts for the maximum amount of variation in the original data set, whereas the maximum amount of the remaining differences is accounted for by PC2, etc. The complexity of the data thereby is greatly reduced, and it may be sufficient to consider only a few PCs to describe gross variations between the included spectra rather than all individual peaks separately. For each PC, the PCA provides scores for every spectrum (cf. signal intensities) and loadings, which specifies the contribution of each individual peak to the PC. Thus, the scores specify the degree of similarity between spectra, whereas loadings indicate peaks that vary in a correlated way between spectra.

**Figure 4:**
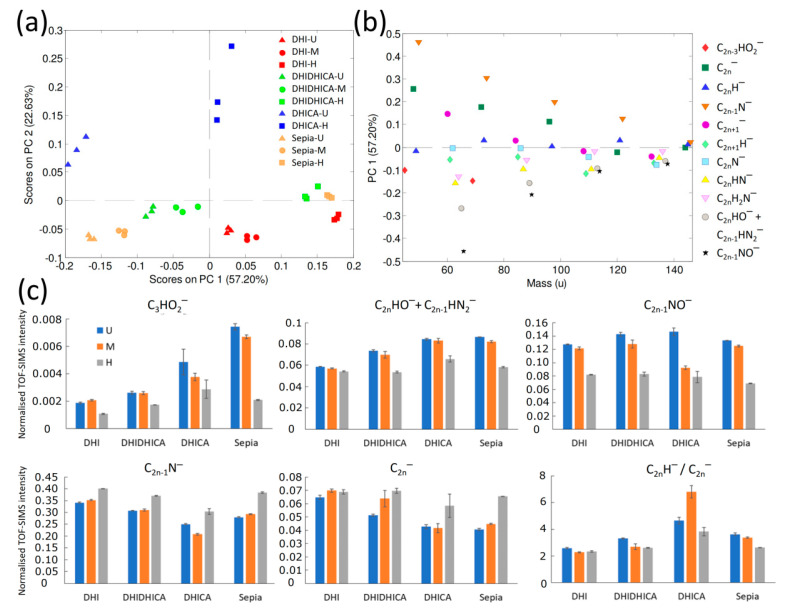
ToF-SIMS signal intensity analysis of negative ions in spectra derived from a selection of eumelanin samples. (**a**) Principal component analysis (PCA) score plot of spectra from DHI (red), DHI + DHICA (1:1) (green), DHICA (blue) and *Sepia* (orange) eumelanin; untreated samples (triangles), and samples after mild (circles) and harsh (squares) maturation, respectively. (**b**) PC1 loadings for peaks assigned to the ion categories indicated in the right-hand list (see also Table 2 for specific ion assignments and Supplementary Materials Figure S2 for PC2 loadings). (**c**) Added peak intensities of ions of some selected categories. The diagrams show mean values with error bars corresponding to ±1 standard deviation (N = 3). Abbreviations: U, untreated; M, mild treatment (100 °C/100 bar for 24 h); H, harsh treatment (250 °C/200 bar for 72 h).

Results of a PCA that included all samples except the DHICA melanin after mild maturation (see below) demonstrated clear spectral variations between the analyzed pigments (Figure 4a,b). Spectra obtained from the same sample are located closely together in the score plot, while those from different samples are clearly separated in chemospace. Furthermore, the spectra from DHI (red symbols), DHI + DHICA (1:1) (green) and *Sepia* (orange) eumelanin are mainly distributed along the (horizontal) PC1 axis. Here, the harshly matured samples are gathered on the right side of the plot (high PC1 scores), whereas spectra from the untreated samples are distinctly separated at lower PC1 scores (left side). For the untreated eumelanins, *Sepia* displays the lowest PC1 scores, DHI the highest, and DHI + DHICA (1:1) occupies an intermediate position. After mild maturation, all spectra are shifted slightly to higher PC1 scores relative to those of the corresponding untreated samples; i.e., in the direction towards the harshly matured samples. These observations indicate clear spectral differences between the untreated DHI, DHI + DHICA (1:1) and *Sepia* eumelanins, small but significant changes upon mild maturation, and similar but more extensive alterations upon harsh treatment, after which only small/negligible differences occur between the various eumelanin types.

The spectra acquired from the DHICA eumelanin do not follow the same pattern as the other three pigment types, since they display higher PC2 scores and no co-localization with the other melanin samples after harsh maturation. This indicates different molecular characteristics of the DHICA melanin compared to the other samples. It has indeed been noted that small variations in the DHI-to-DHICA ratio can have profound effects on the resulting melanins [17]. The inability of the DHICA polymer to adopt any planar π-delocalized structures (see Figure 1 and introduction) could have a significant influence on the fragmentation pattern, which in the end may influence the PCA analysis. Nonetheless, the general effect of harsh treatment is comparable to that of the other analyzed eumelanin types; i.e., a considerable increase in PC1 scores. Furthermore, mildly matured DHICA eumelanin was excluded in the PCA presented in Figure 4a,b because spectra from this particular sample were noticeably different from those of the other pigments (resulting in a PCA that was largely dominated by the spectral differences between this and the other samples). The cause(s) for this marked deviation in spectral characteristics of the mildly treated DHICA eumelanin remains unclear, but most likely is related to contaminant artefacts.

The PC1 loadings provide details about how the spectra differ and how the analyzed eumelanins are affected by maturation (Figure 4b). Because maturation generally leads to increased PC1 scores (Figure 4a), the effect of maturation includes increased signal intensity from ions with high/positive PC1 loadings and decreased intensity from ions with low/negative PC1 loadings. Interestingly, the PC1 loadings (Figure 4b) reveal a systematic pattern in that ions belonging to the same category show similar behavior. Specifically, C_2n−1_N^−^ and C_2n_^−^ ions generally have high/positive PC1 loadings (indicating increased signal intensities upon maturation), whereas C_2n−1_NO^−^ and C_2n_HO^−^ + C_2n−1_HN_2_^−^ ions show low/negative PC1 loadings (indicating lower intensities, consistent with loss of oxygen, but not nitrogen, upon maturation). Furthermore, the different PC1 scores for the untreated eumelanin samples indicate a higher oxygen content in the DHI + DHICA (1:1) eumelanin relative to the DHI eumelanin, which is consistent with the molecular structures of the DHI and DHICA units (Figure 1a). Notably though, an even higher oxygen content occurs in the *Sepia* eumelanin. The relationship between negative PC1 scores and high oxygen content is further corroborated by the negative PC1 loadings for the CHO_2_^−^ and C_3_HO_2_^−^ ions (Figure 4b).

Our observations of the PCA results are verified by the diagrams presented in Figure 4c, which compare the added signal intensities for selected ion categories between the various eumelanin samples. For example, the reduced signal intensities in the O-containing ion categories (Figure 4c, upper row) upon maturation are evident, as well as the differences between the untreated eumelanin samples. There is also a tendency of decreased hydrogen content upon maturation, as revealed by the lower signal intensity ratio of the C_2n_H^−^ to C_2n_^−^ ions. Furthermore, the fact that the observed differences in signal intensities are relatively minor confirms the limited spectral variations between the different samples. Finally, the somewhat aberrant data for the mildly treated DHICA eumelanin are consistent with the PCA (see above), and further emphasize the peculiar nature of this sample.

## 3. Discussion

The results of this study demonstrate that both AHPO and ToF-SIMS are capable of detecting different eumelanins and monitoring molecular changes caused by artificial maturation at elevated temperatures and pressures, thereby making both methods suitable for the analysis of eumelanin residues in multimillion-year-old fossils. Furthermore, both methods have advantages and disadvantages that, to a large extent, complement each other. For example, ToF-SIMS provides spatially resolved molecular information that renders it possible to link molecular eumelanin signals to microstructures on a sample surface, whereas AHPO is capable of verifying and quantifying the eumelanin content in a fossil. Thus, a combination of these techniques allows confident identification and characterization of eumelanin traces in fossilized organismal remains.

The AHPO analysis shows clear and consistent results upon mild maturation, indicating increased cross-linking (increased PTeCA yield) and partial conversion of DHICA via decarboxylation (decreased PTCA yields). After harsh maturation, yields from all three eumelanin markers are considerably reduced, in particular for PTCA, indicating extensive molecular modifications of the DHI and DHICA units into structures that no longer produce these characteristic eumelanin markers during AHPO analysis. However, since PTeCA decreases less than PTCA, harsh maturation leads to an increased PTeCA/PTCA ratio for all melanins studied in this work. A PTeCA/PTCA ratio with a value greater than one, sometimes up to five, is characteristic of fossil eumelanins, because this ratio reflects the degree of cross-linking [4,6,23,25,26,34]. Thus, the harsh maturation experiments are, in this regard, consistent with fossilized eumelanins fossil melanin.

In contrast, the ToF-SIMS spectra show that certain characteristic eumelanin features are retained even after harsh maturation, although significant changes in relative yields of the major peaks were observed. These results indicate that ToF-SIMS is less sensitive than AHPO to molecular modifications that occur upon experimental maturation. For example, the ToF-SIMS spectra are remarkably insensitive to the extensive decarboxylation of the DHICA units that is inferred from our AHPO results. However, an important observation is that eumelanin is clearly recognized in the ToF-SIMS spectra also after harsh treatment; i.e., after maturation-induced molecular changes that cause radical reductions in the yields of eumelanin markers.

The relatively minor effects of artificial maturation on ToF-SIMS spectra of eumelanins, as observed in this work, are consistent with previous studies of fossil squid ink sacs by AHPO and ToF-SIMS [23,25]. AHPO showed significant eumelanin content in fossil ink from two different sources, but the concentration was considerably lower (by a factor of as much as 10^3^) in an ink sample from the Jurassic Posidonia Shale of Germany when compared against a similar, even older (approximately 190 million years) ink sample from the Dorset coast of England. The main difference between these samples were rationalized in terms of extensive cross-linking in the Posidonia Shale ink due to differences in diagenetic conditions [23]. In contrast, ToF-SIMS analysis of similar fossil ink samples showed clear spectral characteristics of eumelanin in both cases, indicating a considerably smaller difference between the two samples [25]. Although ToF-SIMS spectra of the fossil ink from Dorset indicated significantly better preserved eumelanin, in agreement with the AHPO results, the retained "eumelanin features" in the ToF-SIMS spectra of the Posidonia Shale ink sac suggest the presence of eumelanin-specific structures at considerably higher concentrations than indicated by AHPO. Furthermore, our PCA results show that ToF-SIMS spectra of eumelanins after harsh maturation are relatively similar for the different pigment types (i.e., DHI, DHI + DHICA (1:1) and *Sepia*). In turn, these results suggest that harsh maturation may lead to the formation of a stable, eumelanin-related structure that does not produce appreciable yields of the characteristic eumelanin markers upon AHPO treatment, and that such structure may provide the main contribution to the characteristic eumelanin features in the ToF-SIMS spectra of the Posidonia Shale ink sac sample.

Given that harsh maturation was found to cause molecular changes that dramatically reduce the yields of the eumelanin-specific markers during AHPO analysis, while simultaneously retaining the eumelanin-characteristic features in ToF-SIMS, the question arises to what degree the molecular structure can be modified and/or to what extent the ToF-SIMS spectrum can be altered before it is no longer possible to identify a sample as eumelanin, or, in fossils, as originating from eumelanin endogenous to the extinct organism. Regarding the molecular structure, the sample can no longer be assigned to eumelanin if the structure has been changed into something that could also be formed by degradation/diagenetic maturation of other biomolecular species. Clearly, most organic materials will eventually transform into aliphatic and/or aromatic hydrocarbons (kerogens), at which point assignment to the original molecular species is no longer possible. However, it is also possible that maturation leads to the formation of a relatively stable intermediate structure that could still be specific for eumelanin. Similarly, eumelanin identification by ToF-SIMS is no longer possible when the spectrum has been modified to such extent that also other compounds can generate these same spectral features, before or after degradation/diagenetic maturation. Despite previous extensive investigations of related compounds, including, e.g., various types of melanins, porphyrins and other pigments, we have not been able to reproduce the characteristic spectral features of eumelanin in ToF-SIMS spectra of any other compound. In addition, identification of eumelanin in fossils using ToF-SIMS (based on the strict requirements described by Lindgren et al., 2012, [7]) hitherto has always been verified by other techniques (e.g., SEM, TEM, AHPO and/or Fourier transform infrared absorption spectrometry (FTIR)), and in no case have "eumelanin-characteristic" ToF-SIMS spectra been in conflict with these other methods. The present results, i.e., extensive loss of eumelanin signatures during AHPO analysis but retention of characteristics in ToF-SIMS data, are thus consistent with the existence of a robust matured eumelanin molecular structure that does not produce characteristic degradation products upon AHPO treatment, yet still generates mass spectra that can be considered representative of this pigment.

A critical factor for reliable eumelanin identification in fossils is the presence of the N-and NO-containing ions characteristic for this pigment (Table 2), since these exclude the possibility that the spectra are generated by aliphatic and aromatic hydrocarbons (which are typically the main organic materials in carbonized fossils [4]). However, any N- and NO-containing organic must still be considered as a potential source of these ions, including compounds formed during degradation of proteins, such as N-heterocyclic compounds [35] and porphyrins. Whereas porphyrins (heme) have been observed in fossils and shown to be distinguishable from eumelanin by ToF-SIMS [5], protein-related degradation products warrant further investigation. Thus, although previous results indicate that the characteristic eumelanin features in ToF-SIMS spectra also are specific for eumelanin, identification in fossils by ToF-SIMS should, as far as possible, be verified also by other techniques, including AHPO.

The results in this study consistently show increased signal intensity of the C_2n−1_N^−^ ions upon harsh maturation (Figure 4c), to suggest that nitrogen is preserved in the eumelanin structure. This observation is in contrast to previous eumelanin spectra obtained from fossil samples [25], which are generally characterized by reduced signal intensities of both N- and NO-containing ions relative to modern eumelanin references. This in turn indicates that diagenetic maturation is associated with loss of nitrogen and oxygen from the eumelanin structure. Thus, our results demonstrate that artificial maturation, as performed here, does not accurately reproduce all aspects of diagenetic maturation of melanins. Specifically, the maturation protocols applied in this study do not seem capable of reproducing those molecular processes leading to loss of organic nitrogen, something that fossils apparently have been subjected to during natural diagenetic processes.

While details of the molecular changes involved in eumelanin maturation cannot be conclusively resolved from the present results, some relevant information can still be obtained. Clearly, the strong decrease in the yields of all eumelanin-related AHPO markers upon harsh maturation shows that changes, in addition to those that lead to the generation of PDCA (decarboxylation of DHICA) and PTeCA (cross-linking in the 3-position), have occurred. Furthermore, although the ToF-SIMS spectra suggest that the general eumelanin structure is retained after harsh maturation, alterations in peak intensities indicate loss of oxygen and, to some extent, hydrogen (but not nitrogen) from the eumelanin molecular structure. However, approximately 50% of the change in PTCA concentration occurs upon mild maturation, whereas the ToF-SIMS spectra show only minor deviations upon mild maturation and the major changes occur upon harsh maturation. This lack of correspondence between the PTCA concentration after AHPO and yields of the O-containing ions in ToF-SIMS suggest that they reflect different changes in molecular structure upon maturation. For example, loss of oxygen, as indicated by ToF-SIMS, may be associated with loss of the OH-groups at the 5- and 6-positions (Figure 1), whereas the reduced PTCA concentrations upon AHPO may be associated with decarboxylation. The loss of OH-groups at the 5- and 6-positions by pyrolytic dehydration [36] would likely lead to a reduction in the yields of all the AHPO markers (as observed), since that would render the aromatic ring less susceptible to oxidative degradation by hydrogen peroxide. These observations are also incompatible with desorption of physisorbed or chemisorbed water molecules [36], since these should not influence PTCA yields in AHPO or formation of C_n_HO_x_ or C_n_NO fragments in our ToF-SIMS analysis. Alternatively, the results may indicate that mild maturation involves reaction of the DHICA carboxyl groups (including cross-linking), other than complete decarboxylation, and that harsh maturation leads to additional reactions that may include loss of oxygen. Cross-linking upon maturation at position 3 in the DHI or DHICA monomer units, as indicated by the increased yields of PTeCA in our AHPO analysis, is associated with reduced hydrogen content, just as any oxidative cross-linking reaction that may occur during maturation. Thus, formation of cross-links in the eumelanin structure is consistent with the reduced intensity of C_2n_H^−^ ions, as compared to the corresponding C_2n_^−^ ions, observed in the ToF-SIMS spectra upon maturation. The retention of nitrogen, as observed by ToF-SIMS, is opposite to what has been observed for mass-spectroscopy coupled thermogravimetric measurements of natural and synthetic DOPA melanins, where ammonia is produced even at low temperatures [36]. However, these melanins likely contain primary amino groups (from residual proteins or non-cyclized DOPA units) that more easily are lost during pyrolysis.

The deviating ToF-SIMS spectra of DHICA melanin (in particular, the mildly matured sample) may have been caused by contamination, which likely contributed to the signal intensity of the eumelanin-related peaks and thereby distorted the spectra. AHPO is not sensitive to intrusive compounds in a similar manner, as contamination does not contribute to the measured concentrations of the eumelanin-specific markers. The AHPO results for DHICA melanin should therefore not be considered with the same caveats as the ToF-SIMS results for this particular sample. This difference between the techniques also highlights an important limitation of the use of ToF-SIMS when identifying eumelanin, namely, whereby the pigment must be present at relatively high concentrations on the sample surface, at least relative to other organic compounds that may otherwise contribute to the signal intensity of the eumelanin-characteristic fragment ions. However, due to the spatial resolution of the ToF-SIMS measurements, relatively pure eumelanin needs to be present on the surface only in very small structures; i.e., in a sufficiently large surface area to produce a ToF-SIMS spectrum characteristic for eumelanin (about 10 µm^2^) [7].

## 4. Materials and Methods

### 4.1. Materials

Synthetic melanin polymers were prepared according to previously published protocols [37] via tyrosinase-catalyzed oxidation of DHI, DHICA, or a 1:1 mixture of these two monomers (in the following text referred to as DHI, DHICA and DHI:DHICA melanin, respectively). Tyrosinase from mushroom, melanin from *Sepia officinalis* and all other chemicals, unless noted otherwise, were purchased from Sigma-Aldrich (St. Louis, MO, USA). The *Sepia* melanin was washed with ultrapure water (Merck Milli-Q, Sweden, Solna) and ethanol (Rectapur 96%, VWR, Spånga, Sweden) five times each, and dried under vacuum before use. Argon gas (AGA/Linde Sweden, Solna, Sweden, laboratory grade 99.9997%) was used during the maturation experiments.

### 4.2. Experimental Maturation at High Temperatures and Pressures

The two experimental maturation conditions used in this study were selected to emulate conditions experienced by fossils during diagenesis, but also to speed up chemical processes. Previous maturation studies of synthetic melanins [18] were conducted at 100 °C under atmospheric pressure, followed by analysis using AHPO. Pressure is usually considered to be of secondary importance during chemical maturation (relative to temperature), at least under anhydrous conditions [38,39]. Nonetheless, previous experiments involving natural melanins have frequently been conducted at high pressures and temperatures (>200 °C, >200 bar), and analyzed either by electron microscopy [31] or a combination of microscopy and ToF-SIMS [10]. Hence, we chose conditions that facilitate direct comparisons with previous studies of synthetic and natural melanins.

Eumelanin samples used in our maturation experiments (Table 1) were placed and weighed in aluminum foil capsules (Korff AG, Schwitzerland, Oberbipp), which were then sealed by folding the foil multiple times. The capsules were placed in a stainless-steel high-pressure vessel (30 × 10 mm column, Applied Porous Technologies, Tariffville, CT, USA) connected to an argon gas cylinder. The vessel was purged three times by filling it to 80 bar with argon gas and then relieving the pressure through an exhaust pipe. The vessel was filled to about 75% of the desired final pressure, and then heated in a thermostat-controlled oven (GC oven, 5890 series II, Hewlett Packard, CA, USA) to the final temperature. After thermal equilibration for a few minutes, the pressure was adjusted to the final value. Experimental treatments employed in this work included keeping the samples at 100 °C and 100 bar for 24 h or 250 °C and 200 bar for 72 h (hereafter referred to as “mild” and “harsh” maturation, respectively). After this procedure, the capsules were allowed to cool in an argon atmosphere; they were then weighed, and the extracted samples visually inspected.

### 4.3. ToF-SIMS

The eumelanin samples were attached to silicon substrates using double-sided tape. ToF-SIMS analyses were conducted under static SIMS conditions (primary ion dose density <3 × 10^12^ ions/cm^2^) in a ToF-SIMS IV instrument (IONTOF GmbH, Münster, Germany) using 25 keV Bi_3_^+^ primary ions and low-energy electron flooding for charge compensation. Three positive and three negative ion spectra were acquired for each sample, with the ToF-SIMS instrument optimized for high mass resolution (m/Δm 3000–4000). All measurements were repeated by acquiring an additional complete set of data from the same sample batches, with similar results. Only negative ion data are presented herein because the characteristic spectral features for eumelanin are observed only in the negative ion mode. Mass calibration of the negative ion spectra was done using peaks corresponding to the C^−^, C_2_^−^, C_3_^−^, and C_4_^−^ ions.

Principal component analysis (PCA) was performed using the Solo software (version 7.9.5, Eigenvector Inc., Manson, WA, USA), and included all peaks listed in Table 2. The integrated, dead-time corrected signal intensities were normalized to the added signal intensity of all included peaks prior to PCA, and pre-processing included mean centering and Poisson scaling.

### 4.4. Alkaline Hydrogen Peroxide Oxidation (AHPO)

The AHPO was performed as described by Ito et al. 2011 [24]. In brief, 100 µL water suspensions of melanins (containing 0.1 mg DHI melanin, DHICA melanin, DHI + DHICA (1:1) melanin and sepia melanin) were placed in 10-mL screw-capped conical test tubes, to which 375 µL 1 mol/L K_2_CO_3_ and 25 µL 30% H_2_O_2_ were added. The tubes were mixed vigorously at 25 ± 1 °C for 20 h. The reaction was terminated by adding 50 µL 10% Na_2_SO_3_ and the mixture was then added with 140 µL 6M HCl. Reaction mixtures produced by the AHPO treatment were directly subjected to HPLC determination with UV detection [24], employing improved conditions using an ion pair reagent, tetra-*n*-butylammonium bromide [40]. PTCA, PDCA, and PTeCA were analyzed. IsoPTCA was not analyzed because its quantities were much lower than those of PTCA and PTeCA [6,18], thus making this marker less informative.

## 5. Conclusions

Experimental maturation using both high temperatures and pressures substantially alters the eumelanin molecular structure, as evinced by changing yields of PDCA, PTCA, and PTeCA upon AHPO treatment and by systematic changes in our ToF-SIMS spectra. However, whereas AHPO is sensitive to minor molecular alterations, ToF-SIMS can recognize eumelanin even after modifications that lead to exceedingly low yields of typical eumelanin degradation products. After harsh maturation conditions, the ToF-SIMS spectra of the different types of eumelanin (synthetic DHI and DHI + DHICA (1:1) as well as natural *Sepia* melanin) were remarkably similar with respect to their signal intensity distributions of all major eumelanin fragment ions, despite significant differences of these samples prior to maturation; this is consistent with the formation of a stable, eumelanin-specific molecular structure that is largely independent of the specific molecular structure of the original eumelanins, and sufficiently altered to cause radical yield reductions of eumelanin markers upon AHPO treatment. Thus, the results of our study highlight the complementary nature of AHPO and ToF-SIMS when characterizing eumelanin residues in multimillion-year old fossils.

## Figures and Tables

**Table 1:** List of samples used in this study, together with recorded weight losses during our artificial maturation experiments. Abbreviations: U, untreated; M, mild maturation (100 °C/100 bar for 24 h); H, harsh maturation (250 °C/250 bar for 72 h).

	DHI Melanin			DHI + DHICA (1:1) Melanin			DHICA Melanin			*Sepia* Melanin		
Pre-treatment	U	M	H	U	M	H	U	M	H	U	M	H
Weight loss (%)	0	6	14	0	3	18	0	9	20	0	5	47

**Table 2:** Major peaks and ion assignments in negative-ion ToF-SIMS spectra of eumelanins. Observed mass values are given for DHI + DHICA (1:1) eumelanin after harsh experimental maturation, but similar values were observed also for the other samples. Peaks assigned to C_(2n−1)_N^−^ ions are printed in red to highlight the repetitive nature of the spectra.

Observed Mass (*m/z*)	Ion Assignment	Theoretical Mass (*m/z*)
44.999	CHO_2_^−^	44.998
48.001	C_4_^−^	48.000
49.010	C_4_H^−^	49.008
50.006	C_3_N^−^	50.003
60.001	C_5_^−^	60.000
61.008	C_5_H^−^	61.008
62.006	C_4_N^−^	62.003
63.013	C_4_HN^−^	63.011
64.011	C_4_H_2_N^−^ + C_3_N_2_^−^	64.019/64.006
65.013	C_4_HO^−^ + C_3_HN_2_^−^	65.003/65.014
66.000	C_3_NO^−^	65.998
68.999	C_3_HO_2_^−^	68.998
72.000	C_6_^−^	72.000
73.009	C_6_H^−^	73.008
74.006	C_5_N^−^	74.003
84.000	C_7_^−^	84.000
85.007	C_7_H^−^	85.008
86.006	C_6_N^−^	86.003
87.012	C_6_HN^−^	87.011
88.011	C_6_H_2_N^−^ + C_5_N_2_^−^	88.019/88.006
89.014	C_6_HO^−^ + C_5_HN_2_^−^	89.003/89.014
90.002	C_5_NO^−^	89.998
95.998	C_8_^−^	96.000
97.008	C_8_H^−^	97.008
98.004	C_7_N^−^	98.003
107.997	C_9_^−^	108.000
109.005	C_9_H^−^	109.008
110.005	C_8_N^−^	110.003
111.011	C_8_HN^−^	111.011
112.010	C_8_H_2_N^−^ + C_7_N_2_^−^	112.019/112.006
113.014	C_8_HO^−^ + C_7_HN_2_^−^	113.003/113.014
114.009	C_7_NO^−^	113.998
119.994	C_10_^−^	120.000
121.007	C_10_H^−^	121.008
122.002	C_9_N^−^	122.003
131.996	C_11_^−^	132.000
133.002	C_11_H^−^	133.008
134.003	C_10_N^−^	134.003
135.009	C_10_HN^−^	135.011
136.008	C_10_H_2_N^−^ + C_9_N_2_^−^	136.019/136.006
137.013	C_10_HO^−^ + C_9_HN_2_^−^	137.003/137.014
138.013	C_9_NO^−^	137.998
143.992	C_12_^−^	144.000
145.004	C_12_H^−^	145.008
146.001	C_11_N^−^	146.003

## Data Availability

The data presented in this study are available in this article and it’s accompanying supplementary material.

## Supplementary Materials

Supplementary Materials can be found at https://www.mdpi.com/1422-0067/22/1/161/s1. Figure S1. Negative TOF-SIMS spectra of the different eumelanin samples; Figure S2. PC2 loadings from PCA of negative ions in TOF-SIMS spectra of eumelanin samples. Associated scores and PC1 loadings are presented in Figure 4a,b, respectively.

## Abbreviations

AHPO	Alkaline hydrogen peroxide oxidation
ToF-SIMS	Time-of-flight secondary ion mass spectrometry
PCA	Principal component analysis
DHI	5,6-Dihyroxyindole
DHICA	5,6-Dihydroxyindole-2-carboxylic acid
SEM	Scanning electron microscopy
TEM	Transmission electron microscopy
HPLC	High performance liquid chromatography
PDCA	Pyrrole-2,3-dicarboxylic acid
PTCA	Pyrrole-2,3,5-tricarboxylic acid
PTeCA	Pyrrole-2,3,4,5-tetracarboxylic acid
Iso-PTCA	Pyrrole-2,3,4-tricarboxylic acid

## References

[1] Solano F. (2014). Melanins: Skin pigments and much more—Types, structural models, biological functions, and formation routes. New J. Sci..

[2] Simon J.D., Peles D., Wakamatsu K., Ito S. (2009). Current challenges in understanding melanogenesis: Bridging chemistry, biological control, morphology, and function. Pigment Cell Melanoma Res..

[3] D’Ischia M., Wakamatsu K., Cicoira F., Di Mauro E., Garcia-Borron J.C., Commo S., Galván I., Ghanem G., Koike K., Meredith P. (2015). Melanins and melanogenesis: From pigment cells to human health and technological applications. Pigment Cell Melanoma Res..

[4] Lindgren J., Sjövall P., Thiel V., Zheng W., Ito S., Wakamatsu K., Hauff R., Kear B.P., Engdahl A., Alwmark C. (2018). Soft-tissue evidence for homeothermy and crypsis in a Jurassic ichthyosaur. Nature.

[5] Lindgren J., Kuriyama T., Madsen H., Sjövall P., Zheng W., Uvdal P., Engdahl A., Moyer A.E., Gren J.A., Kamezaki N. (2017). Biochemistry and adaptive colouration of an exceptionally preserved juvenile fossil sea turtle. Sci. Rep..

[6] Glass K., Ito S., Wilby P.R., Sota T., Nakamura A., Bowers C.R., Vinther J., Dutta S., Summons R., Briggs D.E.G. (2012). Direct chemical evidence for eumelanin pigment from the Jurassic period. Proc. Natl. Acad. Sci. USA.

[7] Lindgren J., Uvdal P., Sjövall P., Nilsson D.E., Engdahl A., Schultz B.P., Thiel V. (2012). Molecular preservation of the pigment melanin in fossil melanosomes. Nat. Commun..

[8] Carney R.M., Vinther J., Shawkey M.D., D’Alba L., Ackermann J. (2012). New evidence on the colour and nature of the isolated Archaeopteryx feather. Nat. Commun..

[9] Manning P.L., Edwards N.P., Bergmann U., Anné J., Sellers W.I., van Veelen A., Sokaras D., Egerton V.M., Alonso-Mori R., Ignatyev K. (2019). Pheomelanin pigment remnants mapped in fossils of an extinct mammal. Nat. Commun..

[10] Colleary C., Dolocan A., Gardner J., Singh S., Wuttke M., Rabenstein R., Habersetzer J., Schaal S., Feseha M., Clemens M. (2015). Chemical, experimental, and morphological evidence for diagenetically altered melanin in exceptionally preserved fossils. Proc. Natl. Acad. Sci. USA.

[11] Pralea I.-E., Moldovan R.-C., Petrache A.-M., Ilies M., Heghes S.-C., Ielciu I., Nicoară R., Moldovan M., Ene M., Radu M. (2019). From extraction to advanced analytical methods: The challenges of melanin analysis. Int. J. Mol. Sci..

[12] Ito S. (2003). A chemist’s view of melanogenesis. Pigment Cell Res..

[13] Pezzella A., Napolitano A., d’Ischia M., Prota G. (1996). Oxidative polymerisation of 5,6-dihydroxyindole-2-carboxylic acid to melanin: A new insight. Tetrahedron.

[14] D’Ischia M., Napolitano A., Pezzella A., Land E.J., Ramsden C.A., Riley P.A. (2005). 5,6-Dihydroxyindoles and Indole-5,6-diones. Adv. Heterocycl. Chem..

[15] D’Ischia M., Napolitano A., Pezzella A. (2011). 5,6-Dihydroxyindole chemistry: Unexplored opportunities beyond eumelanin. Eur. J. Org. Chem..

[16] Micillo R., Panzella L., Iacomino M., Prampolini G., Cacelli I., Ferretti A., Crescenzi O., Koike K., Napolitano A., d’Ischia M. (2017). Eumelanin broadband absorption develops from aggregation-modulated chromophore interactions under structural and redox control. Sci. Rep..

[17] Micillo R., Panzella L., Koike K., Monfrecola G., Napolitano A., d’Ischia M. (2016). “Fifty shades” of black and red or how carboxyl groups fine tune eumelanin and pheomelanin properties. Int. J. Mol. Sci..

[18] Ito S., Wakamatsu K., Glass K., Simon J.D. (2013). High-performance liquid chromatography estimation of cross-linking of dihydroxyindole moiety in eumelanin. Anal. Biochem..

[19] Panzella L., Gentile G., D’Errico G., Della Vecchia N.F., Errico M.E., Napolitano A., Carfagna C., d’Ischia M. (2013). Atypical structural and π-electron features of a melanin polymer that lead to superior free-radical-scavenging properties. Angew. Chem. Int. Ed..

[20] Wakamatsu K., Ito S. (2002). Advanced chemical methods in melanin determination. Pigment Cell Res..

[21] Chen C.-T., Chuang C., Cao J., Ball V., Ruch D., Buehler M.J. (2014). Excitonic effects from geometric order and disorder explain broadband optical absorption in eumelanin. Nat. Commun..

[22] Pezzella A., Panzella L., Natangelo A., Arzillo M., Napolitano A., d’Ischia M. (2007). 5,6-Dihydroxyindole tetramers with “Anomalous” interunit bonding patterns by oxidative coupling of 5,5′,6,6′-tetrahydroxy-2,7′-biindolyl: Emerging complexities on the way toward an improved model of eumelanin buildup. J. Org. Chem..

[23] Glass K., Ito S., Wilby P.R., Sota T., Nakamura A., Russell Bowers C., Miller K.E., Dutta S., Summons R.E., Briggs D.E.G. (2013). Impact of diagenesis and maturation on the survival of eumelanin in the fossil record. Org. Geochem..

[24] Ito S., Nakanishi Y., Valenzuela R.K., Brilliant M.H., Kolbe L., Wakamatsu K. (2011). Usefulness of alkaline hydrogen peroxide oxidation to analyze eumelanin and pheomelanin in various tissue samples: Application to chemical analysis of human hair melanins. Pigment Cell Melanoma Res..

[25] Lindgren J., Nilsson D.-E., Sjövall P., Jarenmark M., Ito S., Wakamatsu K., Kear B.P., Schultz B.P., Sylvestersen R.L., Madsen H. (2019). Fossil insect eyes shed light on trilobite optics and the arthropod pigment screen. Nature.

[26] Tanaka G., Parker A.R., Hasegawa Y., Siveter D.J., Yamamoto R., Miyashita K., Takahashi Y., Ito S., Wakamatsu K., Mukuda T. (2014). Mineralized rods and cones suggest colour vision in a 300 Myr-old fossil fish. Nat. Commun..

[27] Sanyova J., Cersoy S., Richardin P., Laprévote O., Walter P., Brunelle A. (2011). Unexpected materials in a Rembrandt painting characterized by high spatial resolution cluster-TOF-SIMS imaging. Anal. Chem..

[28] Stephan T., Jessberger E.K., Heiss C.H., Rost D. (2003). TOF-SIMS analysis of polycyclic aromatic hydrocarbons in Allan Hills 84001. Meteorit. Planet. Sci..

[29] Lindgren J., Sjövall P., Carney R.M., Uvdal P., Gren J.A., Dyke G., Schultz B.P., Shawkey M.D., Barnes K.R., Polcyn M.J. (2014). Skin pigmentation provides evidence of convergent melanism in extinct marine reptiles. Nature.

[30] McNamara M.E., van Dongen B.E., Lockyer N.P., Bull I.D., Orr P.J. (2016). Fossilization of melanosomes via sulfurization. Palaeontology.

[31] McNamara M.E., Briggs D.E.G., Orr P.J., Field D.J., Wang Z. (2013). Experimental maturation of feathers: Implications for reconstructions of fossil feather colour. Biol. Lett..

[32] Saitta E.T., Kaye T.G., Vinther J. (2019). Sediment-encased maturation: A novel method for simulating diagenesis in organic fossil preservation. Palaeontology.

[33] Graham D.J., Wagner M.S., Castner D.G. (2006). Information from complexity: Challenges of TOF-SIMS data interpretation. Appl. Surf. Sci..

[34] Pinheiro F.L., Prado G., Ito S., Simon J.D., Wakamatsu K., Anelli L.E., Andrade J.A.F., Glass K. (2019). Chemical characterization of pterosaur melanin challenges color inferences in extinct animals. Sci. Rep..

[35] Wiemann J., Fabbri M., Yang T.-R., Stein K., Sander P.M., Norell M.A., Briggs D.E.G. (2018). Fossilization transforms vertebrate hard tissue proteins into N-heterocyclic polymers. Nat. Commun..

[36] Simonovic B.R., Wilczok T. (1995). Direct evidence of melanin decomposition by simultaneous DTA, TGA and MS analysis. J. Serb. Chem. Soc..

[37] D’Ischia M., Wakamatsu K., Napolitano A., Briganti S., Garcia-Borron J.-C., Kovacs D., Meredith P., Pezzella A., Picardo M., Sarna T. (2013). Melanins and melanogenesis: Methods, standards, protocols. Pigment Cell Melanoma Res..

[38] Landais P., Michels R., Elie M. (1994). Are time and temperature the only constraints to the simulation of organic matter maturation?. Org. Geochem..

[39] Monthioux M., Landais P., Monin J.-C. (1985). Comparison between natural and artificial maturation series of humic coals from the Mahakam delta, Indonesia. Org. Geochem..

[40] Ito S., Del Bino S., Hirobe T., Wakamatsu K. (2020). Improved HPLC conditions to determine eumelanin and pheomelanin contents in biological samples using an ion pair reagent. Int. J. Mol. Sci..

