# Supplementary material for: Chemical Evaluation of Eumelanin Maturation by ToF-SIMS and Alkaline Peroxide Oxidation HPLC Analysis
Source: Int J Mol Sci. 2020 Dec 26;22(1):161. doi: 10.3390/ijms22010161 (PMC7796430; doi:10.3390/ijms22010161)
Supplement: Supplementary file 1 [file ijms-22-00161-s001.pdf]

Supplementary materials
for

Chemical evaluation of eumelanin maturation by ToF-SIMS and alkaline peroxide oxidation HPLC analysis

Martin Jarenmark[1]*, Peter Sjövall[2]*, Shosuke Ito[3], Kazumasa Wakamatsu[3], Johan Lindgren[1]

[1] Department of Geology, Lund University, 223 62 Lund, Sweden.; martin.jarenmark@geol.lu.se; johan.lindgren@geol.lu.se
[2] RISE Research Institutes of Sweden, Materials and Production, 501 15 Borås, Sweden; peter.sjovall@ri.se
[3] Institute for Melanin Chemistry, Fujita Health University, Toyoake, Aichi 470-1192, Japan; sito@fujita-hu.ac.jp; kwaka@fujita-hu.ac.jp
* Correspondence: martin.jarenmark@geol.lu.se; peter.sjovall@ri.se

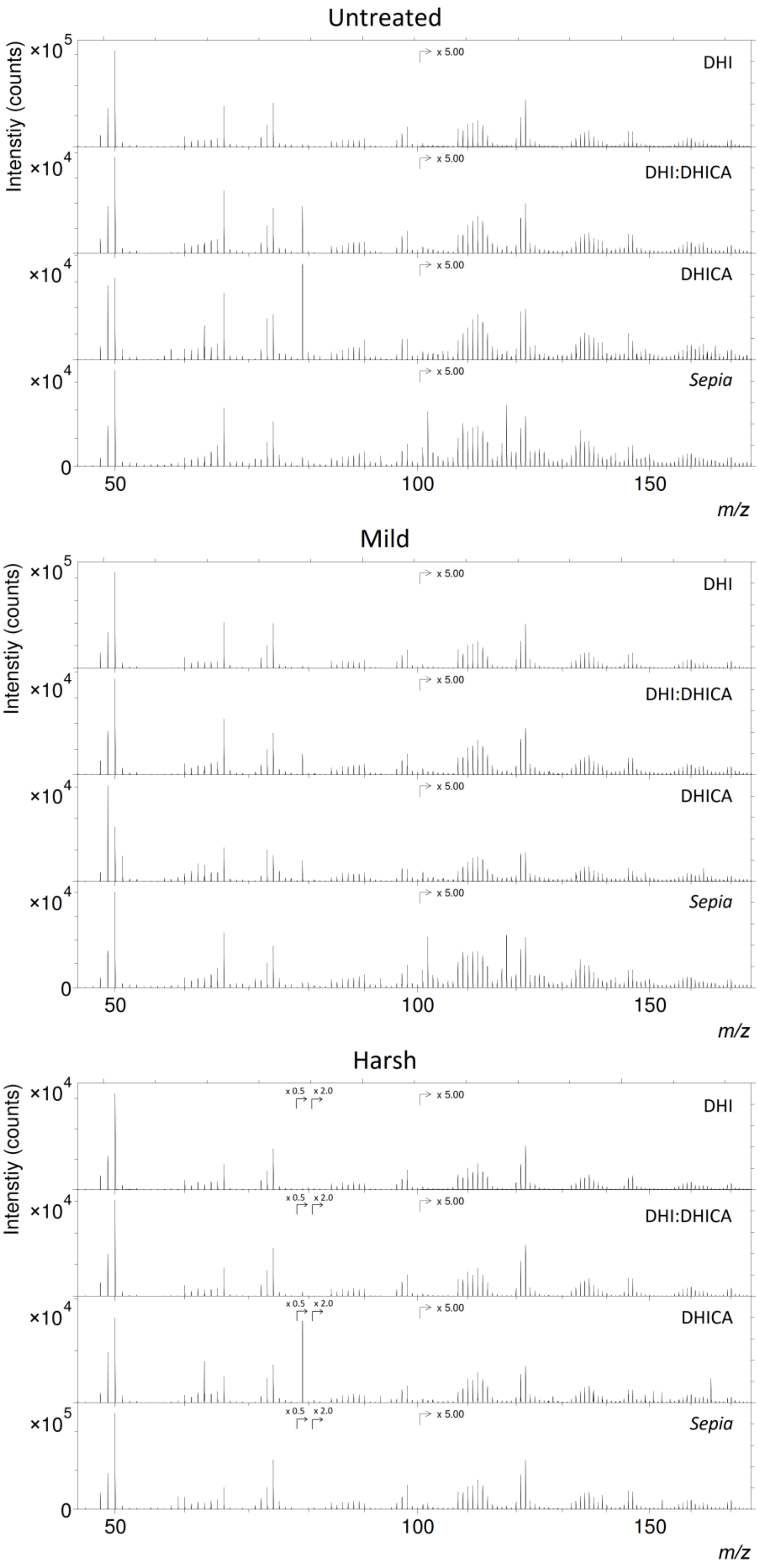

Figure S1. Negative TOF-SIMS spectra of the different eumelanin samples.

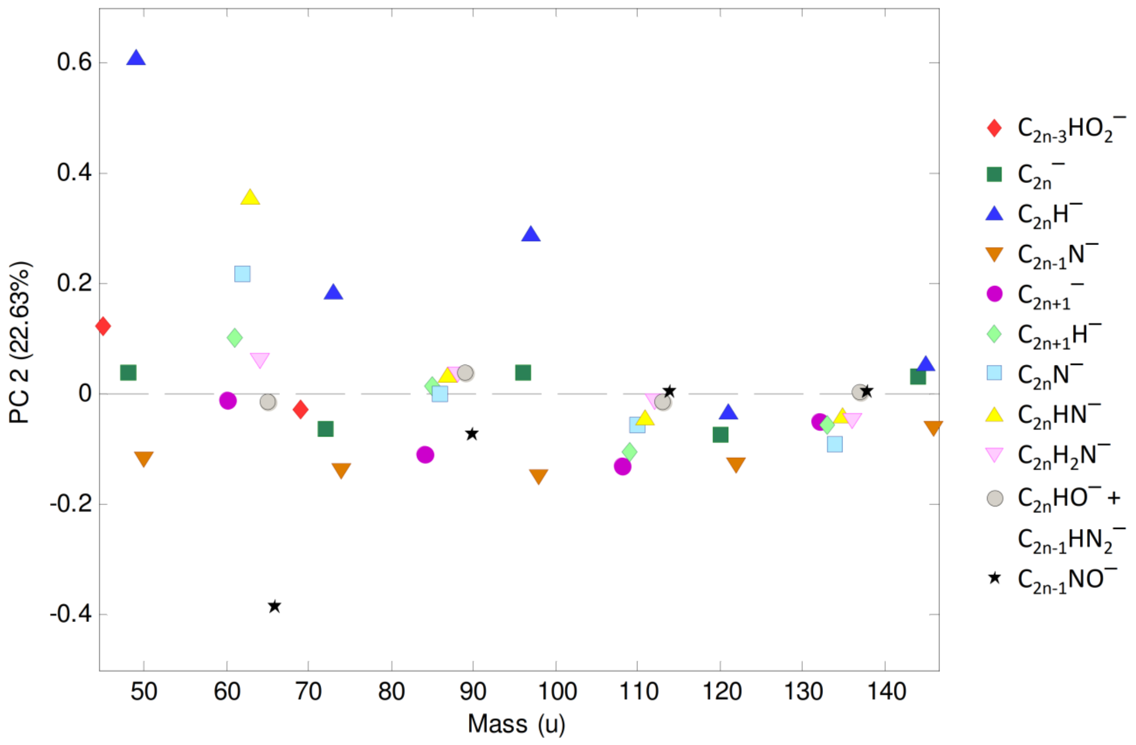

Figure S2. PC2 loadings from PCA of negative ions in TOF-SIMS spectra of eumelanin samples. Associated scores and PC1 loadings are presented in Figure 4a and 4b, respectively.
